# A rare case of numerous parasitic myomas after laparoscopic myomectomy

**DOI:** 10.1111/jog.16187

**Published:** 2024-12-16

**Authors:** Kazuhisa Fujita, Kazuhiko Tsukada, Fumi Utsumi, Kazuhiro Sugihara, Makoto Urano, Kiyosumi Shibata

**Affiliations:** ^1^ Department of Obstetrics and Gynecology Fujita Health University, Bantane Hospital Nagoya Japan; ^2^ Department of Diagnostic Pathology Fujita Health University, Bantane Hospital Nagoya Japan

**Keywords:** leiomyomatosis peritonealis disseminata, morcellation, parasitic myoma, pulmonary benign metastatic leiomyomatosis

## Abstract

Parasitic myoma is a relatively rare disease in which one or more leiomyomas form outside the uterus; however, the detailed causes are unknown. Few sporadic reports are available, and per our research, the maximum number of parasitic myomas reported to date was 26, and almost all cases were treated by surgical resection. We report a rare case of numerous parasitic myomas in the abdominal cavity, possibly including an intrathoracic lesion, which could not be resected completely. The patient was a 42‐year‐old, gravid 2, para 0, artificially aborted 2, and not yet menopausal woman. She had undergone laparoscopic myomectomy at a different hospital 6 years prior. Laparoscopically, numerous hard white masses, ranging from 1 mm to approximately 55 mm in size, were found in the abdominal cavity. The masses were particularly numerous in the omentum and mesentery but were also found on the diaphragm, abdominal peritoneum, and intestinal surface. The patient was pathologically diagnosed with multiple benign leiomyomas. On computed tomography, a similar nodule was observed in the right lower lobe of the lung. Despite using in‐bag morcellation, as in this case, numerous parasitic myomas occurred, suggesting that greater caution should be exercised when explaining laparoscopic myomectomy to patients.

## INTRODUCTION

Parasitic myoma is a relatively rare disease in which one or more leiomyomas develop outside the uterus. A similar disease is leiomyomatosis peritonealis disseminata (LPD), in which multiple leiomyomatous nodules occur in the abdominal cavity. Parasitic myoma and LPD are rare diseases, and the distinction between them is unclear. Therefore, considerable confusion and overlap likely exist in previous reports. Since its publication by Ostrzenski,^1^ few sporadic case reports of parasitic myoma have been published. LPD was first characterized by Willson and Peale in 1952, and approximately 200 cases of LPD have been recorded since then.^2^, ^3^ Many patients have a history of laparoscopic morcellation.^4^ Therefore, parasitic myoma or iatrogenic LPD is thought to occur when myoma tissue fragments that are cut into small pieces by laparoscopic morcellation and scattered into the abdominal cavity receive blood supply and attach. However, spontaneous cases in patients with no history of surgery and those with a history of laparotomy or hysteroscopy rather than laparoscopic surgery have also been reported^5^, ^6^, ^7^, ^8^; therefore, the details of the pathogenesis are unknown.

In a systematic review by Van der Meulen et al. in 2016, 69 cases from 44 reports were extracted, with a mean age at onset of 40.8 ± 7.5 years (range 24–57), a median time from surgery to diagnosis of 48 months (range 1–192), and the number of parasitic myomas of 2.9 ± 3.3 (range 1–16). The incidence of parasitic myoma after laparoscopic uncontained morcellation has been reported to be 0.12%–0.95%.^4^ Although sporadic reports have been published since then, to our knowledge, the maximum number of parasitic myomas was 26,^9^ and almost all cases were treated by surgical resection.^10^, ^11^, ^12^

Here, we report a rare case of numerous parasitic myomas in the abdominal cavity, possibly including an intrathoracic lesion, and complete surgical resection was impossible.

### CASE PRESENTATION

**FIGURE 1:**
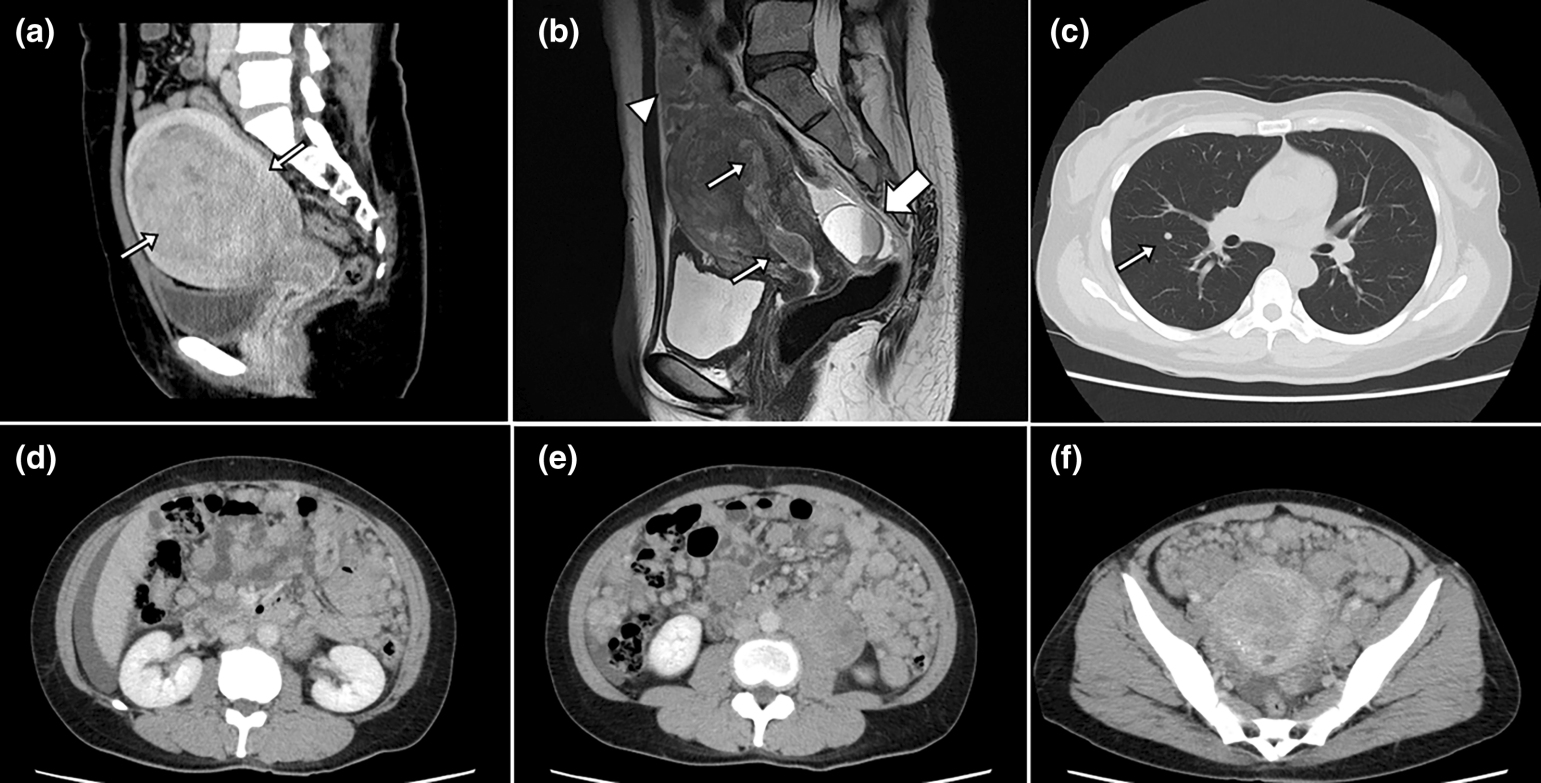
(a) Preoperative contrast‐enhanced computed tomography (CT) images from a laparoscopic myomectomy performed at a different hospital 6 years prior. One uterine myoma is found in the uterine body (arrows). (b) Preoperative T2‐weighted magnetic resonance images (MRI). The uterine body is enlarged, especially in the anterior wall, and many uterine myoma‐like mass lesions are observed in the uterine cavity and muscle layers (thin arrows). Furthermore, many nodular lesions (arrowheads) with contrast effects and mild diffusion restriction are seen in the abdominal cavity on contrast‐enhanced MRI (data not shown). A cystic lesion with a liquid surface formation is observed in the Douglas pouch, suspected to be an endometrial cyst or a peritoneal inclusion cyst (thick arrow). (c–f) Preoperative contrast‐enhanced CT images. Numerous nodular lesions of various sizes with contrast effects and clear borders throughout the entire abdominal area. A similar nodule is also observed in one location in the right lower lobe of the lung (arrow).

We present the case of a 42‐year‐old, gravid 2, para 0, artificially aborted 2, and not yet menopausal woman. She had undergone laparoscopic myomectomy at a different hospital 6 years prior. A preoperative contrast‐enhanced computed tomography (CT) scan is shown in Figure 1a (magnetic resonance imaging [MRI] was likely avoided because of tattoos). According to the previous doctor's surgical records, one intramural myoma measuring 60 mm in diameter and weighing 175 g was removed from the posterior wall of the uterus. The resected myoma was placed into a tissue‐containment bag (MorSafe™) and then removed from the body while being cut into small pieces using a morcellator, with no tissue scattering in the abdominal cavity. The uterine incision was closed with Z‐sutures using size 0 monofilament synthetic absorbable sutures. The operation took 123 min, with a blood loss of 125 mL, and was completed without any complications. Postoperative histopathological examination revealed a uterine leiomyoma without malignancy. The patient was not taking any medications after surgery. Family or lifestyle history was not significant.

The patient presented with menorrhagia and irregular bleeding. Contrast‐enhanced MRI revealed that the uterine body was enlarged, especially in the anterior wall, and many uterine myoma‐like mass lesions were observed in the uterine cavity and muscle layers (Figure 1b, thin arrows). Furthermore, many nodular lesions with contrast effects and mild diffusion restriction were observed in the abdominal cavity (Figure 1b, arrowheads). A cystic lesion with a liquid surface formation was observed in the Douglas pouch, which was suspected to be an endometrial cyst or a peritoneal inclusion cyst (Figure 1b, thick arrow). Contrast‐enhanced CT revealed numerous nodular lesions of various sizes with contrast effects and clear borders throughout the abdominal area, including the omentum and mesentery (Figure 1d–f). A similar nodule was observed in the right lower lobe of the lung (Figure 1c, arrow). Cervical and endometrial cytology results were negative.

Based on these findings and the patient's history of laparoscopic myomectomy, recurrence, dissemination, and metastasis of the uterine myoma were suspected. The patient wanted the treatment to be as minimally invasive as possible. Resection of all numerous nodules completely was thought to be impossible; therefore, diagnostic laparoscopic surgery was performed to confirm the diagnosis and exclude malignant disease. Simultaneously, hysteroscopic surgery was performed to improve the irregular bleeding and menorrhagia.

**FIGURE 2:**
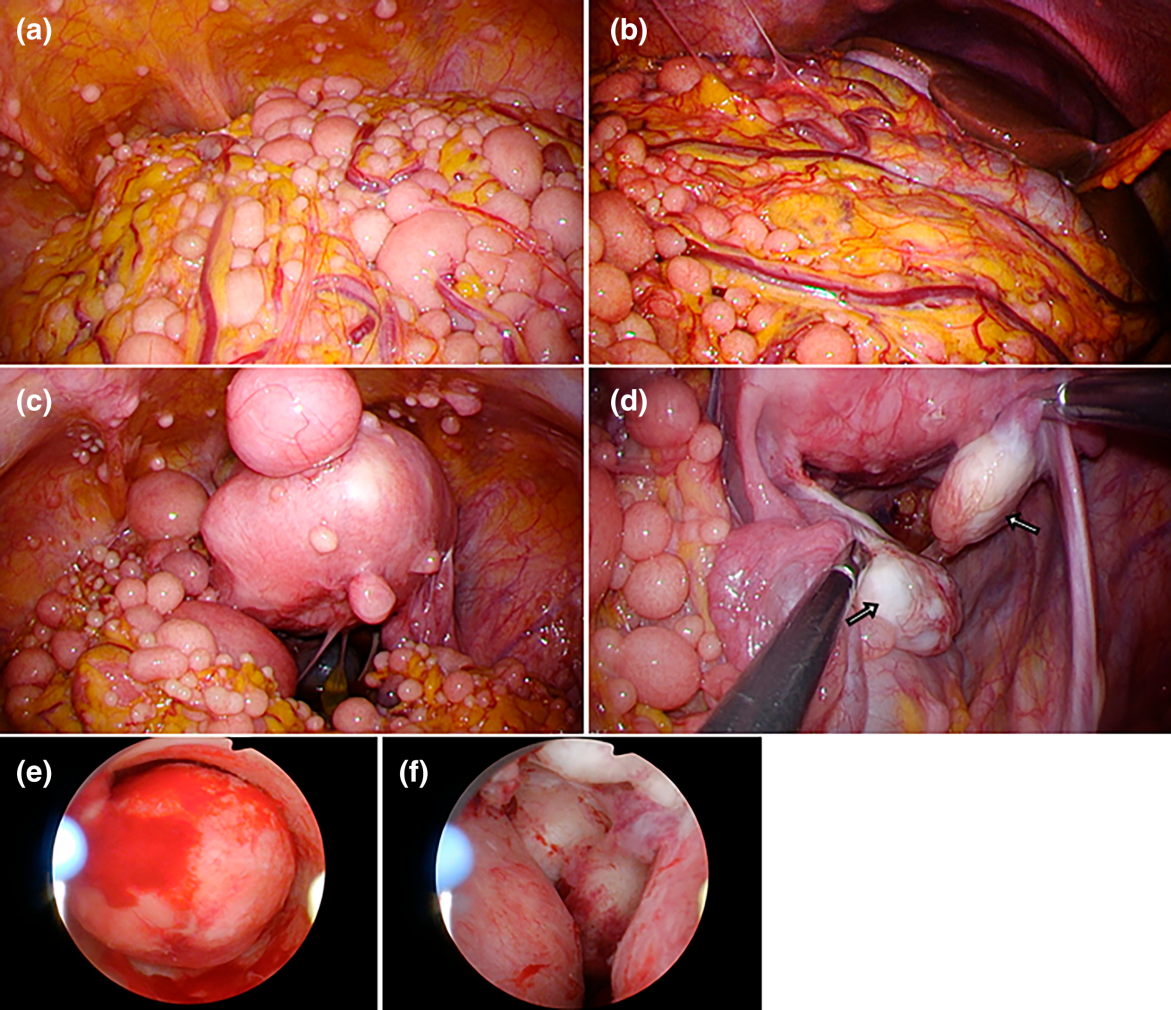
Findings from single‐port laparoscopic and hysteroscopic surgery. (a–d) Numerous hard white masses, ranging from 1 mm to approximately 55 mm in size, were found in the abdominal cavity. (e, f) Several submucosal myomas are observed in the uterine cavity.

Laparoscopic surgery was performed via a single incision in the umbilicus. Numerous hard white masses, ranging from 1 mm to approximately 55 mm in size, were found in the abdominal cavity (Figure 2a–d). The masses were particularly numerous in the omentum and mesentery but were also found on the diaphragm, abdominal peritoneum, and intestinal surface. The uterus was 10 × 6 cm in size, and multiple subserosal myomas were found. Soft membranous adhesions and adhesion cysts were found around the uterus; however, they were easily detachable, and uterine mobility was good (Figure 2c,d). No gross abnormalities were observed in the adnexa on either side (Figure 2d, arrows). Several representative masses were resected as pathological specimens. The pelvic cyst in the Douglas pouch resembled an endometriotic cyst, and no continuity with the ovary was observed; therefore, the cyst was resected. Hysteroscopy revealed several submucosal myomas in the uterine cavity (Figure 2e,f). The protruding masses were excised with a loop electrode until the uterine cavity was flattened, and a total of 13 myomas were removed.

**FIGURE 3:**
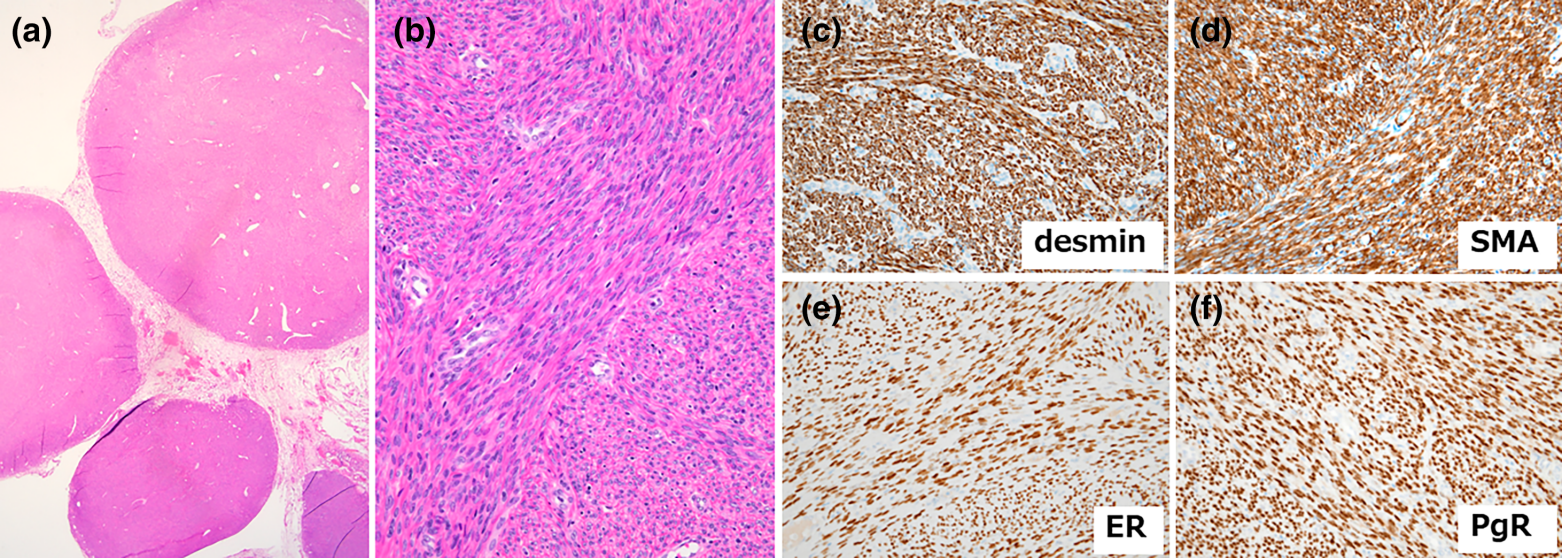
Histopathological findings. (a) Multiple solid nodules attached to the peritoneal adipose tissue (×1.25). (b) Spindle‐shaped cells with bland nuclei arranged in fascicles and bundles (×20). (c–f) Immunohistochemical findings. The tumor cells are positive for desmin (c), smooth muscle actin (d), estrogen receptor (e), and progesterone receptor (f). (×20).

Postoperative histopathological examination revealed multiple intraperitoneal (Figure 3a) and submucosal nodules, both with the same characteristics. Microscopically, the nodules were composed of spindle‐shaped cells arranged in fascicles and bundles. The tumor cells had cytologically bland, uniform nuclei with fine chromatin and small nucleoli (Figure 3b). Immunohistochemical staining showed that the tumor cells were positive for desmin, smooth muscle actin, estrogen receptor, and progesterone receptor (Figure 3c–f) and focally positive for PAX8. The Ki‐67 labeling index was low (<5%). The patient was diagnosed with multiple benign leiomyomas. A pelvic cyst in the Douglas pouch showed evidence of endometriosis without atypia. No malignancy was observed in any of the surgical specimens.

A gonadotropin‐releasing hormone (GnRH) antagonist was administered for 6 months after the surgery. Her chief complaints of menorrhagia and irregular bleeding improved. Contrast‐enhanced CT, performed 6 months later, showed a decrease in the number of nodules and a reduction in their size. The nodule in the lung also shrank. From 6 months later, a dienogest will be administered, and follow‐up observations will be performed every 3 months.

The patient included in this study provided written informed consent for the publication of their clinical data, and this study conforms to the provisions of the Declaration of Helsinki.

## DISCUSSION

As mentioned above, the maximum number of reported parasitic myomas is 26,^9^ and almost all cases are treated with surgical resection.^10^, ^11^, ^12^ However, this case had numerous parasitic myomas in the abdominal cavity, possibly including an intrathoracic lesion, which could not be completely resected.

Parasitic myoma or iatrogenic LPD is thought to occur when myoma tissue fragments, scattered into the abdominal cavity by laparoscopic morcellation, receive a blood supply and attach. The patient in this case had a history of laparoscopic myomectomy at another hospital 6 years prior. This surgery was performed using a tissue‐containment bag, known as in‐bag morcellation. Regarding the use of laparoscopic morcellators, the US Food and Drug Administration (FDA) issued a safety notice in 2014, stating that when performing laparoscopic hysterectomy or myomectomy using an electric morcellator in women with uterine myomas, there is a risk of seeding unexpected cancer tissue, especially uterine sarcoma, into the abdominal cavity. Currently, we do not recommend the use of electric morcellators for these surgeries. Furthermore, in December 2020, the FDA recommended against using electric morcellators is contraindicated in patients aged >50 years or postmenopausal cases, and that a tissue‐containment bag must be used when using an electric morcellator. Many studies recommend in‐bag morcellation to prevent parasitic myomas.^9^ However, the fact that, even when using in‐bag morcellation, as in this case, numerous parasitic myomas can occur suggests that greater caution should be exercised when explaining laparoscopic myomectomy to patients.

The incidence of parasitic myoma after laparoscopic uncontained morcellation is very low (0.12%–0.95%); the use of a morcellator does not necessarily lead to parasitic myoma.^4^ In addition, in this case, although in‐bag morcellation was performed to prevent tissue scattering, numerous myomas were generated, along with possibly intrathoracic lesions. Although the intrathoracic lesion has not been histologically proven, it is likely to be a myoma, as it has shrunk with the administration of a GnRH antagonist, just like the intraperitoneal lesion. Considering these facts, the cause of parasitic myoma cannot be explained solely by the scattering of myoma tissue fragments into the abdominal cavity. Intravenous leiomyomatosis (IVL) and pulmonary benign metastatic leiomyomatosis (PBML) are diseases that cause benign leiomyomas outside the uterus, such as parasitic myomas or LPD.^13^ These diseases can occur regardless of whether a patient has a history of laparoscopic surgery. The cause of these diseases is assumed to be hematogenous or lymphatic metastasis of uterine leiomyoma; however, some are also thought to be tumors derived from vascular smooth muscle cells.^14^ Another theory is that it is caused by metaplasia of submesothelial multipotent mesenchymal cells.^15^ The present case was suspected to be related to these diseases. However, the diagnostic boundaries between parasitic myoma and LPD, IVL and PBML are unclear; therefore, considerable confusion or overlap is likely present in previous reports. In any case, there are likely unknown factors on the patient's side that allow hematogenous or lymphatic metastasis and dissemination of myoma, and further unknown factors allowing myoma to attach to other organs. However, these factors have not yet been elucidated, and further research is required.

In the present case, complete surgical resection was deemed impossible, and only a tumor biopsy was performed using diagnostic laparoscopy, raising concerns regarding future tumor growth. Takeda et al. found that parasitic myomas have progesterone receptors,^16^ and that the parasitic myoma they observed grew rapidly after pregnancy,^17^ suggesting that sex steroid hormones are involved in the growth of parasitic myomas. In this case, the tumor cells were positive for estrogen and progesterone receptors; therefore, a GnRH antagonist was selected to prevent tumor progression. Although GnRH antagonists have reduced the number and size of the nodules, side effects mean that GnRH antagonists cannot be continued indefinitely. If sequential dienogest therapy fails to control the disease, bilateral adnexal resection may be necessary.

## CONCLUSION

We observed a rare case of numerous parasitic myomas in the abdominal cavity, possibly including intrathoracic lesions, and complete surgical resection was impossible. The fact that, even when using in‐bag morcellation, as in this case, numerous parasitic myomas could occur suggests that greater caution should be exercised when explaining laparoscopic myomectomy to patients.

## AUTHOR CONTRIBUTIONS

**Kazuhisa Fujita:** Conceptualization; data curation; investigation; methodology; project administration; validation; visualization; writing – original draft; writing – review and editing. **Kazuhiko Tsukada:** Conceptualization; data curation; supervision. **Fumi Utsumi:** Conceptualization; data curation; formal analysis; supervision; writing – review and editing. **Kazuhiro Sugihara:** Conceptualization; supervision. **Makoto Urano:** Data curation; supervision; visualization; writing – review and editing. **Kiyosumi Shibata:** Conceptualization; data curation; project administration; supervision; writing – review and editing.

## CONFLICT OF INTEREST STATEMENT

The authors declare no conflicts of interest.

## Data Availability

Data sharing not applicable to this article as no datasets were generated or analyzed during the current study.
